# Predicting Early Dropout in a Digital Tobacco Cessation Intervention: Replication and Extension Study

**DOI:** 10.2196/54248

**Published:** 2024-11-27

**Authors:** Linda Q Yu, Michael S Amato, George D Papandonatos, Sarah Cha, Amanda L Graham

**Affiliations:** 1 Innovations Center Truth Initiative Washington, DC United States; 2 Department of Medicine Mayo Clinic College of Medicine and Science Rochester, MN United States; 3 Center for Statistical Sciences School of Public Health Brown University Providence, RI United States; 4 Department of Oncology Georgetown University Medical Center Washington, DC United States

**Keywords:** digital interventions, attrition, engagement, dropout, tobacco, smoking, cessation, mobile health, internet, mobile phone

## Abstract

**Background:**

Detecting early dropout from digital interventions is crucial for developing strategies to enhance user retention and improve health-related behavioral outcomes. Bricker and colleagues proposed a single metric that accurately predicted early dropout from 4 digital tobacco cessation interventions based on log-in data in the initial week after registration. Generalization of this method to additional interventions and modalities would strengthen confidence in the approach and facilitate additional research drawing on it to increase user retention.

**Objective:**

This study had two research questions (RQ): RQ1—can the study by Bricker and colleagues be replicated using data from a large-scale observational, multimodal intervention to predict early dropout? and RQ2—can first-week engagement patterns identify users at the greatest risk for early dropout, to inform development of potential “rescue” interventions?

**Methods:**

Data from web users were drawn from EX, a freely available, multimodal digital intervention for tobacco cessation (N=70,265). First-week engagement was operationalized as any website page views or SMS text message responses within 1 week after registration. Early dropout was defined as having no subsequent engagement after that initial week through 1 year. First, a multivariate regression model was used to predict early dropout. Model predictors were dichotomous measures of engagement in each of the initial 6 days (days 2-7) following registration (day 1). Next, 6 univariate regression models were compared in terms of their discrimination ability to predict early dropout. The sole predictor of each model was a dichotomous measure of whether users had reengaged with the intervention by a particular day of the first week (calculated separately for each of 2-7 days).

**Results:**

For RQ1, the area under the receiver operating characteristic curve (AUC) of the multivariate model in predicting dropout after 1 week was 0.72 (95% CI 0.71-0.73), which was within the range of AUC metrics found in the study by Bricker and colleagues. For RQ2, the AUCs of the univariate models increased with each successive day until day 4 (0.66, 95% CI 0.65-0.67). The sensitivity of the models decreased (range 0.79-0.59) and the specificity increased (range 0.48-0.73) with each successive day.

**Conclusions:**

This study provides independent validation of the use of first-week engagement to predict early dropout, demonstrating that the method generalizes across intervention modalities and engagement metrics. As digital intervention researchers continue to address the challenges of low engagement and early dropout, these results suggest that first-week engagement is a useful construct with predictive validity that is robust across interventions and definitions. Future research should explore the applicability and efficiency of this model to develop interventions to increase retention and improve health behavioral outcomes.

## Introduction

A well-established challenge for digital health behavior change interventions is that many users disengage before achieving a meaningful dose of treatment necessary for desired outcomes [1-5]. A recent paper by Bricker et al [6] aimed to identify early markers of dropout that could generalize across platforms and aid the design of rescue interventions to mitigate dropout. Using the data from 2 clinical trials of 4 web- or app-based tobacco cessation platforms, they compared a variety of classification models to predict early dropout. They considered predictors including baseline and demographic variables, as well as daily log-in data from the first 7 days after registration. The best-performing model that predicted whether a user dropped out after the initial week, across all platforms, was a logistic regression model using daily log-in count data from the first 7 days. This method has the advantage of being straightforward and easy to implement for researchers and intervention designers. The finding that baseline and demographic variables did not contribute to greater predictive accuracy means the same approach can be used for both robust clinical trial datasets and observational datasets that may contain less information. Bricker et al [6] noted that it remains to be seen whether the high predictive power of this model can translate to other platforms and study designs like real-world datasets, which are much more heterogeneous compared to clinical trial data.

This study aimed to conceptually replicate and extend Bricker et al [6] by applying the best-performing model from their analyses to a large, real-world dataset from a tobacco cessation intervention that includes both web and SMS text messaging components. While Bricker et al [6] conceived of the log-in measure as a single, unifying measure across digital platforms, it is exclusively web or app based and does not account for the multimodal nature of many tobacco cessation interventions. In particular, SMS text messaging interventions have been shown to be effective at increasing abstinence outcomes in smoking cessation [7-9] and are now commonly deployed alongside or integrated with web interventions. The log-in measure may also not be practical for web-based platforms in some cases. Given these technical considerations, this study aimed to extend the work of Bricker et al [6] to a multimodal intervention with a broader set of engagement measures beyond log-in data.

The value of predicting early dropout is the opportunity to prevent it. To guide such targeted intervention efforts, we sought to characterize the time course of early engagement and its relationship with early dropout. In particular, we measured how rapidly the proportion of users who remain engaged decreases after registration and then compared the classification performance of early dropout models using engagement data at different time points. Performance metrics of those models can inform decisions about the most efficient timing to intervene for users at risk of early dropout. Such decisions are not universally conclusive but rather require consideration of trade-offs between the costs of a potential rescue intervention and the pool of potential recipients. For example, it might be more efficient to deploy a low-cost notification to a larger group of users with lower average risk, whereas a more resource-intensive appeal might be more efficiently deployed to a smaller group of users that are at high risk of dropping out.

To examine the practical implications of predicting and preventing early dropout, we leveraged a sizable, multiyear dataset from a real-world digital tobacco cessation intervention that incorporates web and SMS text messaging components. The research questions (RQs) addressed by this study were twofold: RQ1—can the model in Bricker et al [6] be replicated in a large-scale, observational, and multimodal dataset to predict early dropout? and RQ2—can patterns of engagement in the first week be used to identify which users are at the greatest risk of early dropout, for the development of potential “rescue” interventions? By answering this pair of RQs, this study aimed to demonstrate a simple, generalizable approach that can be used to address the persistent issue of early dropout across a broad range of digital interventions.

## Methods

### Study Design

This study uses observational analysis of data from EX, a free, multimodal digital tobacco cessation intervention developed by Truth Initiative in collaboration with the Mayo Clinic. Results are reported according to STROBE (Strengthening the Reporting of Observational studies in Epidemiology) recommendations [10].

### Setting

EX is a multimodal, evidence-based tobacco cessation program designed around the US Public Health Service tobacco dependence treatment clinical practice guidelines [11], Social Cognitive Theory [12], and the Mayo Clinic model for engaging tobacco users in cessation treatment [13]. As a real-world intervention, EX has evolved throughout the study period, although the theoretical approach and behavior change techniques have remained consistent. EX educates tobacco users about the behavioral and physiological aspects of nicotine addiction and helps them build coping skills and strategies for quitting through individually tailored content, hands-on exercises, and videos. It also includes the longest-running, internet-based social network dedicated to tobacco cessation where users can connect with current and former tobacco users for real-time support (EX Community).

The website is fully integrated with a bidirectional and dynamically tailored SMS text message program that users can opt into during website registration. The 12-week SMS text message program is tailored around a number of characteristics, including a user’s quit date, their primary tobacco product, and patterns of engagement with the website. Content includes tips and encouragement, quotes excerpted from the EX Community, links to the EX website, as well as prompts for the user to text back a quit date, their motivations for quitting, the name of a supportive friend or family member, and on-demand keywords for further assistance. Email support is also tailored to each individual, designed to drive ongoing engagement.

Roughly 3000 tobacco users register on EX each month. Most users come through Google ads or organic searches and are motivated to quit or actively engaged in the quitting process. A nominal advertising spend has been consistent over the past decade.

### Ethical Considerations

This study was reviewed and determined to be exempt by the Advarra institutional review board (Pro00082535). To register on EX, individuals must agree to its terms of use and privacy policy, which grants permission for their data to be used in observational research to advance the science of nicotine dependence treatment. As such, no additional screening information was obtained, and no separate informed consent was solicited. Users were not compensated for this research. All study data were deidentified prior to analysis. Truth Initiative has stringent privacy and security policies, which include a comprehensive privacy policy, strong encryption used in the transmission of all health-related information, electronic access controls on all personally identifiable information, and industry-standard network protection and intrusion detection.

### Participants

We examined users who registered between September 1, 2016, and April 13, 2022 (N=143,121). In this study, adult tobacco users aged 18 years or older who opted into the SMS text message program for whom website page view data were available (unavailable for ~2.5% of users who likely used do-not-track software) were included in the analyses. These criteria yielded 70,265 participants.

### Data Sources

#### Baseline Characteristics

Available measures from website registration used to characterize the study sample included gender, age, tobacco use frequency (every day, some days, and not at all), and zip code (mapped to Rural Area Continuum Codes per Amato and Graham [14]).

#### Engagement Data

User activity was tracked for 1 year after registration. Website page views were recorded with Adobe Analytics, and SMS text message responses were recorded in EX’s custom SMS text messaging application database.

The engagement was operationalized as any website page views or SMS text message responses. Early dropout was defined as having no engagement after day 7 after registration. Under this definition, 38.6% (27,118/70,265) of users were categorized as dropouts. For comparison, the proportion of early dropouts ranged between 49.27% and 65.32% in the interventions analyzed by Bricker et al [6].

### Statistical Analyses

First, descriptive statistics were used to determine the risk of early dropout and to characterize the sample using data drawn from website registration. To address RQ1, daily engagement on days 2-7 (1 binary variable for each day) was examined as a predictor of early dropout in a modified Poisson regression model with a logarithmic link and robust error variance [15]. All 6 engagement variables were entered into the model simultaneously. A value of 1 was assigned to each day if the engagement count was positive and 0 otherwise. Of note, all study participants had at least 1 page view on the day they registered on the website. Therefore, day 1 in this model is confounded with the intercept and represents the risk of early dropout among study participants who failed to reengage with the intervention in the first week after their initial enrollment. Regression coefficients for days 2-7 represent the relative risk of early dropout among participants engaging with the study on a particular day relative to those who failed to reengage in the first week after enrollment, controlling for their engagement pattern on the remaining days of that first week. Our statistical model thus provides a modified replication of that used by Bricker et al [6], using a dichotomized, multimodal definition of engagement for the predictors.

To address RQ2, we used modified Poisson regression with robust SEs to examine 6 distinct patterns of engagement as predictors of early dropout, with the goal of informing potential “rescue” interventions. In each univariate regression model, the sole predictor of dropout was “return engagement,” a dichotomous measure of whether someone had returned to the website by a particular day of the first week (days 2-7). For example, an individual who reengaged on day 3 but then disengaged from the intervention again from days 4 to 7 would have a positive value starting from the day 3 model through day 7, because the models consider such a person a “re-engager by Day 3.” Running a series of day-specific models against each other allowed us to obtain accuracy and performance metrics for the effect of delaying a hypothetical rescue intervention by each additional day and to compare the models against each other. By definition, the intercept in these models corresponds to subjects that were coded as 0 on that particular day, that is, those failing to reengage by that day of the week.

Both models used 80% (56,238/70,265) of the data for training purposes and 20% (14,027/70,265) of the data to test performance, with a single 80:20 split stratified by month to evenly represent users from across the study period. Predictive performance metrics included area under the receiver operating characteristic curve (AUC), sensitivity, specificity, positive predictive value, negative predictive value, and Brier score (mean squared prediction error) as calculated on the training set and evaluated on the test set. For comparison under different intervention cost trade-off scenarios, metrics were evaluated under classification cutoffs of 0.5 and 0.3. AUCs were compared against each other with the *roc.test* function in the R library *pROC* [16].

## Results

### Participants

The analytic sample included 70,265 participants. The baseline characteristics of the sample are presented in Table 1.

**Table 1:** Baseline characteristics of the analytic sample (N=70,625).

			Values, n (%)
**Age** **group (years)**			
	18-24	8060 (11.5)	
	25-30	15,650 (22.3)	
	31-44	24,611 (35.0)	
	45-64	19,736 (28.1)	
	Older than 65	2208 (3.1)	
**Sex**			
	Male	21,695 (30.9)	
	Female	48,084 (68.4)	
	Other or not reported	486 (0.7)	
**Tobacco** **use**			
	Every day	68,617 (97.7)	
	Some days	1586 (2.2)	
**Metro** **areas**			
	Large metro	29,154 (41.5)	
	Small metro	23,442 (33.3)	
	Rural	12,405 (17.6)	

### Patterns of Engagement

Engagement rates by day during the first week after registration are shown in Table 2. The daily engagement metrics (dichotomized as any engagement vs none) are relevant to RQ1, whereas the cumulative reengagement metrics are relevant to RQ2. The latter declined more rapidly than the former, with daily reengagement rates diminishing to single digits by day 4. Overall, 36.7% (27,174/70,265) of EX users did not reengage with the intervention during the initial week after registration. The first day of return engagement was most commonly day 2 (26,796/70,265, 38.1%) followed by a smaller proportion on day 3 (9613/70,265, 13.7%). Day 4 and onward saw single-digit percentages of those returning for the first time following registration. The cumulative return numbers on each day reflect the sum of the first day of return numbers over previous days.

**Table 2:** Number and proportion of engaged users in the initial week after registration under different definitions of daily intervention engagement (N=70,265).

	Day 1^a^, n (%)	Day 2, n (%)	Day 3, n (%)	Day 4, n (%)	Day 5, n (%)	Day 6, n (%)	Day 7, n (%)
Daily engagement	70,265 (100)	26,796 (38.1)	20,838 (29.7)	15,947 (22.7)	12,138 (17.3)	10,844 (15.4)	10,073 (14.3)
First day of return engagement	N/A^b^	26,796 (38.1)	9613 (13.7)	3146 (4.5)	1498 (2.1)	1154 (1.6)	884 (1.3)
Cumulative return engagement	N/A	26,796 (38.1)	36,409 (51.8)	39,555 (56.3)	41,053 (58.4)	42,207 (60.1)	43,091 (61.3)

^a^Day 1=program enrollment (all users engage by definition).

^b^N/A: not applicable.

### RQ1: Can the Model in Bricker et al [6] be Replicated in a Large-Scale Observational, Multimodal Dataset to Predict Early Dropout?

An examination of the risk ratios for early dropout based on a daily engagement model showed that those who failed to reengage with the intervention during the first week after registration had a 60% risk of not reengaging with the intervention at any further point during the study period (ie, “dropped out”; see Table 3). For those who returned for even a single day during that week, the early dropout risk was reduced monotonically with time, with the magnitude of reduction ranging from 24% (relative risk=0.76, 95% CI 0.74-0.78) for those who returned on day 3 to 55% (relative risk=0.45, 95% CI 0.42-0.57) for those who returned on day 7.

**Table 3:** Relative risk (95% CI) of early dropout under a daily engagement model.

Predictor	Relative risk coefficient (95% CI)
Intercept^a^	0.60 (0.59-0.61)
Day 2	0.60 (0.59-0.62)
Day 3	0.76 (0.74-0.78)
Day 4	0.59 (0.57-0.61)
Day 5	0.64 (0.61-0.67)
Day 6	0.57 (0.54-0.60)
Day 7	0.45 (0.42-0.57)

^a^The intercept corresponds to the risk of early dropout for study participants who failed to reengage with the intervention on days 2-7 of the initial week.

The predictive performance metrics for the daily engagement model were evaluated in an unbiased manner in our 20% test set and are outlined in Table 4. The discrimination ability of the model (AUC=0.72, 95% CI 0.71-0.73) considerably exceeded the 0.50 value expected by chance and was comparable to that of similar models reported in Bricker et al [6] (AUC range=0.60-0.94). As expected, the sensitivity and negative predictive value of the model decreased, while the specificity and positive predictive value increased, when the classification threshold was changed from 0.3 to 0.5. The Brier score of the model was 0.20, below the 0.25 value expected by chance, indicating adequate calibration.

**Table 4:** Test set performance metrics of first-week daily engagement model in predicting early dropout (no engagement after day 7).

Model performance metric	Classification threshold=0.30	Classification threshold=0.50
Sensitivity	0.86	0.59
Specificity	0.49	0.73
PPV^a^	0.50	0.57
NPV^b^	0.84	0.74
True positive, n	4566	3123
True negative, n	4195	6342
False positive, n	4495	2348
False negative, n	771	2214

^a^PPV: positive predictive value.

^b^NPV: negative predictive value.

### RQ2: Can Patterns of First-Week Engagement be Used to Identify Which Users are at the Greatest Risk of Early Dropout to Inform Potential “Rescue” Interventions?

The 6 cumulative return engagement models examined the effect of delayed return by each additional day in the first week on dropout. A comparison of the model AUCs shows that the prediction accuracy increases until day 4 (AUC=0.66), and levels off thereafter (Table 5). Two other prevalence-free model performance metrics (calculated under a classification probability of 0.3) give a more detailed picture—as the number of days that it takes a user to reengage with the intervention increases, specificity increases but sensitivity decreases. In other words, with each successive day we wait until making a judgment call about the likelihood of early dropout, there is a reduction in the number of false positives (ie, users identified as likely to drop out early who do not actually do so), at the cost of an increase in the number of false negatives (ie, users identified as likely to remain engaged with the intervention who actually drop out early).

**Table 5:** Test set performance comparisons of the 6 cumulative return models in predicting early dropout (no engagement after day 7) at classification threshold=0.3.

	Day 2	Day 3	Day 4	Day 5	Day 6	Day 7
AUC^a^ test (95% CI)	0.63 (0.62-0.64)	0.65 (0.64-0.66)	0.66 (0.65-0.67)	0.66 (0.65-0.67)	0.66 (0.65-0.67)	0.66 (0.65-0.67)
Sensitivity	0.79	0.67	0.64	0.62	0.60	0.59
Specificity	0.48	0.63	0.68	0.70	0.72	0.73
PPV^b^	0.48	0.53	0.55	0.56	0.57	0.57
NPV^c^	0.78	0.76	0.75	0.75	0.75	0.74
True positive, n	4197	3602	3408	3292	3204	3123
True negative, n	4135	5481	5941	6117	6249	6342
False positive, n	4555	3209	2749	2573	2441	2348
False negative, n	1140	1735	2045	2601	2133	2214

^a^AUC: area under the receiver operating characteristic curve.

^b^PPV: positive predictive value.

^c^NPV: negative predictive value.

## Discussion

### Principal Findings

This study examined the relationship between first-week engagement and early dropout in a large observational dataset from a real-world digital tobacco cessation program. We drew on the methods in Bricker et al [6] to address two RQs: RQ1—does first-week engagement predict early dropout in a large-scale, observational dataset of a multimodal (web and SMS text message) digital tobacco cessation intervention? and RQ2—can first-week engagement be used to inform rescue interventions to prevent dropout? With regard to RQ1, we found that a dichotomized measure of engagement that encompassed web and SMS text message use predicted early dropout with acceptable accuracy [17]. The ability of first-week engagement to predict early dropout was considerably above chance (AUC=0.72) and within the range of AUC values described for the interventions in Bricker et al [6], providing independent validation of their method. With regard to RQ2, reengagement with the intervention any day in the first-week after registration predicted a lower likelihood of early dropout than no reengagement at all, with model predictive performance metrics suggesting that different parts of the week (days 2-3 vs days 4-7) traded off in terms of accurately identifying the users who would drop out.

There were several methodological differences between this study and Bricker et al [6]. First, this study used an observational dataset drawn from a real-world intervention over a 5.5-year time period instead of study participants in a time-limited randomized controlled trial. These characteristics strengthen the external validity of our study and may result in greater heterogeneity in participant characteristics, behavior, and the intervention itself as it evolved throughout the years. Another difference is that 1-week dropout outcomes in this study were defined as users who had no further activity (across both web and SMS text message) after this time period, whereas the previous paper had used groups defined by functional clustering methods that allowed some activity after the first week [18]. Our definition is relatively simpler to implement without the need for sophisticated methods and yielded comparable model performance. Finally, EX is a web and SMS text message intervention, which may differ in reengagement and notification strategies from the app- and web-based platforms examined in Bricker et al [6]. Taken together, the models in this study performed with satisfactory accuracy and within the range of the model performances in Bricker et al [6], suggesting that this method is generalizable across platforms, populations, and measures of engagement.

A salient question during the design of a potential rescue intervention to curb dropout is when to intervene. Ideally, one would like to identify users at risk of dropping out as early as possible. However, it is important to consider the trade-offs, taking into consideration the performance of each additional day in the week to predict dropouts with the cost of a possible intervention. For example, if an intervention is a very low cost (eg, sending out an email or push notification), then a model that includes the days with higher sensitivity is desirable in order to target anyone identified as a predicted dropout. A greater number of false positives (ie, users identified as early dropouts who did not do so) is acceptable since the downsides are few, although there may be opportunity costs to delivering a rescue intervention versus other treatment content. However, if the intervention is more costly (eg, proactive human outreach), then a model including the days with higher specificity (lower false positives) would be more suitable. A holistic approach balancing the trade-offs, alongside factors such as user tolerance for notifications, can inform intervention development. As the model comparison we outlined here is easily implementable and generalizable, researchers using other types of digital interventions can apply it to their platform to characterize their own patterns of user reengagement.

### Strengths and Limitations

The strengths of this study included the large sample size of users over multiple years from a freely available intervention and the 1 year of follow-up data. We used rigorous methods for classification, including 80:20 performance testing and 2 classification thresholds. A limitation of the study is the limited amount of demographic data, although Bricker et al [6] showed that the inclusion of baseline characteristics did not improve model prediction performance. The AUC in the first-week daily engagement model was lower than the best model predictions for 3 of the 4 interventions in the original study (0.94-0.85), but higher than 1 (0.60). The lower performance metrics in this study are likely due to the more heterogenous sample in our study compared to clinical trial datasets but were still within an acceptable range. For the cumulative return models, the performance was notably lower (AUCs=0.63-0.66). These models should be interpreted with caution—performance metrics, such as sensitivity and specificity, should be taken into account with other considerations when designing a rescue intervention, as discussed.

### Future Directions

An important next step in this research is to examine factors that may mediate the relationship between continued engagement and health outcomes. Bricker et al [18] showed that users who continued to engage for a month or more were more likely to quit smoking. However, self-selection to continue engagement in that study and other observational studies challenges causal attribution. Future research using alternative designs should further investigate the causal impact of continued engagement on abstinence accounting for potentially confounding factors, such as motivation or confidence.

Previous studies, including those based on the EX intervention [8], have demonstrated the effectiveness of sending tailored reminders in increasing engagement [19-21]. In general, rescue interventions should be in the service of increasing the user’s intrinsic motivation and self-efficacy, which have been shown to be associated with retention [22-25]. For example, a reminder notification could point the user toward community support or information about quitting medications [26-29]. Any single strategy likely is not sufficient. People who use digital interventions for behavior change drop out for many reasons [30], not the least of which is limited time and competing priorities from other aspects of their lives [5,31]. Thus, the intervention must be seen as useful, relevant, and important in furthering their goals for users to make accessing the intervention a priority [32].

### Conclusions

In conclusion, we independently validated the method outlined in Bricker et al [6] using first-week engagement to predict dropout in a large, observational dataset from a multimodal digital intervention for tobacco cessation. Additionally, a model based on the rate of cumulative return engagement over the first week can serve as a promising and easily implementable method for comparing and developing interventions to prevent dropout as early as practically possible.

## Abbreviations

AUC: area under the receiver operating characteristic curve
RQ: research question
STROBE: Strengthening the Reporting of Observational studies in Epidemiology

## Data Availability

The datasets generated and analyzed during this study are available from the corresponding author on reasonable request with a data sharing agreement with Truth Initiative.
